# Sunscreen Use among a Population of Saudi University Students

**DOI:** 10.1155/2020/4732721

**Published:** 2020-03-16

**Authors:** Mohammed I. AlJasser, Abdullah Aljumah, Mohannad Alzaydi, Abdullah Alassaf, Suliman Alassafi, Maha T. Alassafi, Mohammed Almedlej, Emad Masuadi

**Affiliations:** ^1^College of Medicine, King Saud Bin Abdulaziz University for Health Sciences, Riyadh, Saudi Arabia; ^2^Division of Dermatology, Ministry of National Guard Health Affairs, Riyadh, Saudi Arabia; ^3^King Abdullah International Medical Research Center, Riyadh, Saudi Arabia

## Abstract

**Introduction:**

Sunscreen is an important method of sun protection. Many studies were conducted worldwide on the use of sunscreen but only few done in Saudi Arabia. The aim of our study is to assess the prevalence, practices, and factors associated with sunscreen use among Saudi university students.

**Materials and Methods:**

A cross-sectional study was performed at King Saud bin Abdulaziz University for Health Sciences in Riyadh, Saudi Arabia. A questionnaire on the use of sunscreen was created in English. Quota sampling technique was used since the sample was divided according to gender and college year.

**Results:**

A total of 1,011 students were enrolled. Approximately half were males (*n* = 510). Half of the students used sunscreen (*n* = 515, 51%). Female gender, high family income, previous history of sunburn, tanning bed use, and use of other sun protection methods were factors independently associated with sunscreen use. The main reasons for using sunscreen were prevention of sunburns, dark spots, skin cancer, and overall skin darkening. Eighty percent of participants used other methods of sun protection. Sunscreen with a sun protection factor (SPF) > 30 was used in 59% of students. However, the majority did not know if the sunscreen they use provided broad-spectrum coverage or not. Only 35% of students apply sunscreen in both sunny and cloudy days. Most students apply sunscreen less than 10 minutes before going out and do not repeat the application throughout the day. More than 90% of students seem to apply insufficient amount of sunscreen.

**Conclusion:**

Almost half of the population in the study use sunscreen. We have identified several areas of improper use of sunscreen. Increasing the awareness of effective sunscreen use in our community might be needed.

## 1. Introduction

Sun exposure has both beneficial and harmful effects as the main source of vitamin D and a major cause of skin cancer, respectively [1]. Ultraviolet (UV) radiation from the sun is the main cause of these effects. One of the main methods to protect the skin from the harmful effects of UV radiation is the application of sunscreen. Sunscreen is a chemical product made to protect the skin from UV radiation [2]. Sunscreen has been shown to reduce the development of melanoma and nonmelanoma skin cancer [3, 4].

The climate in Saudi Arabia is mostly sunny throughout the year with a UV index reaching 12 in the months from April to August [5]. Many studies have been conducted worldwide on sun exposure and sun protection practices including sunscreen use. However, there are only a few studies which addressed sunscreen use in Saudi Arabia [6–8]. Those studies estimated that 8–35% of the population use sunscreen. University students constituted a major portion of those who use sunscreen [7, 8]. The aim of our study is to assess sunscreen use among Saudi university students.

Previous local studies were mainly conducted to assess knowledge and awareness regarding sun exposure. Sunscreen use was included as one of the several aspects of those studies. The current study focused mainly on aspects related to the details of sunscreen use such as reasons and factors associated with use, method of application, and characteristics of the sunscreen used. The use of other sun protection methods, including clothing that is important in the Saudi culture (such as shemagh and niqab), was also assessed. Knowing more details about sunscreen use among Saudi university students will help in conducting community educational campaigns and future research related to the effect of UV on Middle Eastern skin type.

## 2. Methods

The study has been performed according to the Declaration of Helsinki principles and was approved by the institutional ethics committee at King Abdullah International Medical Research Center (IRBC/872/16). This cross-sectional study was conducted at King Saud bin Abdulaziz University for Health Sciences in Riyadh, Saudi Arabia. The university has seven colleges specialized in health sciences with approximately 3,790 registered students. Male and female students from different colleges were included in the study.

Sample size was calculated based on the previously reported 8% prevalence of sunscreen use in Saudi Arabia [6]. The online calculator Roasoft (http://www.raosoft.com/samplesize.html) was used. With a population of 3,790 students, a margin of error of 1.5%, and a confidence level of 95%, the required sample size was 944. Taking into account an approximately 10% nonresponse rate, a sample size of 1,040 was targeted. Quota sampling technique was used since the sample was divided according to gender and college year. The population was first equally divided into male and female subgroups, and then an equal proportion was taken from each academic year. Informed consent was obtained from all enrolled students.

Questionnaire forms were created in English as all students at the university are proficient in English. The questionnaire was assessed by two dermatologists for content validity. Next, the questionnaire was piloted on 60 students. The final questionnaire included the following sections: demographics, general sunscreen use, method of sunscreen application, type of sunscreen, sun exposure, and skin cancer.

Data analysis was done using the statistical package for social science version 20 (SPSS In., Chicago, IL, US). Descriptive statistics including frequencies and percentages were used to describe categorical variables such as gender and college year. Mean and standard deviation were generated for numerical data such as age. Univariate and multivariate logistic regression analyses were performed to assess factors associated with sunscreen use. A test with a *p* value of ≤0.05 was considered statistically significant.

## 3. Results

A total of 1,011 students were enrolled. Approximately half of the population were males (*n* = 510) and in junior years (*n* = 530) (Table 1). The mean age of the population was 21 ± 2 years. Most of our population (71%) had skin types 3 and 4.

Sunscreen was found to be used by 51% (*n* = 515) of the students (Table 2). In univariate analysis, factors associated with sunscreen use included female gender, high family income, previous history of sunburn, use of tanning beds, and use of other sun protection methods (Table 2). The association of sunscreen use with all the aforementioned factors continued to be statistically significant after performing multivariate logistic regression analysis (Table 2). There was no statistically significant difference in sunscreen use between junior and senior students. Females were approximately 10 times more likely to use sunscreen than males (OR 10.038, *P* value < 0.001). There was a trend towards more likelihood of sunscreen use in students who had more sunburns in the past. Students with >3 sunburns in the past were approximately 5 times more likely to use sunscreen (OR 5.373, *P* value < 0.001). Tanning bed users were more likely to use sunscreen (OR 2.430, *P* value 0.043). Sunscreen use was more in those who use other sun protection methods (OR 1.779, *P* value 0.009).

**Figure 1:**
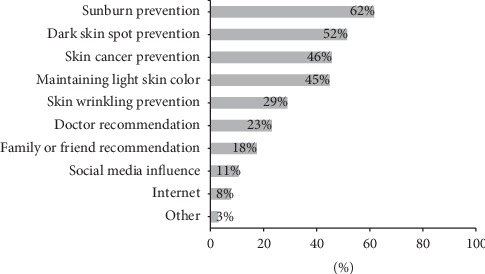
Reasons for using sunscreen (*n* = 515).

**Figure 2:**
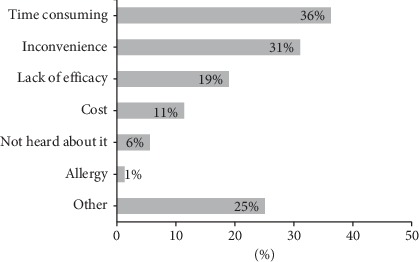
Reasons for not using sunscreen (*n* = 496).

**Figure 3:**
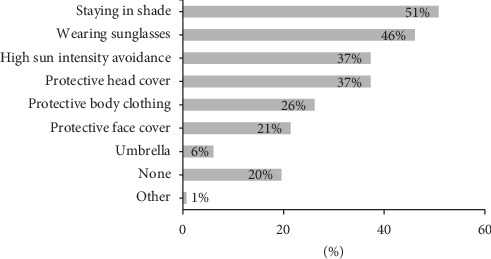
Prevalence of the use of other methods for sun protection (*n* = 1,011).

Students used sunscreen mainly to prevent sunburns (62%), dark spots (51%), and skin cancer (46%) (Figure 1). Almost half of them also used sunscreen to maintain an overall light skin color. The most common reasons for not using sunscreen were time consumption (36%), inconvenience (31%), and lack of efficacy (19%) (Figure 2). Other reasons for not using sunscreen included cost issues (11%) and not knowing about sunscreen (6%). The use of sun protection methods other than sunscreen was common (80%) and included staying in shade, wearing sunglasses, avoiding high sun intensity, and protective clothing (such as shemagh and niqab) (Figure 3).

Table 3 summarizes the details of sunscreen use. Almost all students use sunscreen in summer and less than half of them use it other seasons. Only 35% apply sunscreen in both sunny and cloudy days. A majority of students (74%) apply sunscreen both while in Saudi Arabia and when going abroad on vacation. Approximately half (45%) of the students apply sunscreen only when doing outdoor activities. Intentional sun exposure for longer durations when applying sunscreen was stated by 33% of students. More than half (52%) of the students apply sunscreen for less than 10 minutes before going out. The majority (62%) do not repeat the application of sunscreen throughout the day. Fifty percent apply less than quarter teaspoon amount of sunscreen, and 41% apply quarter to half teaspoon. In females, 50% apply sunscreen before wearing makeup.

The sun protection factor (SPF) of the sunscreen was unknown to 28% of students. A sunscreen with SPF >30 was used in 59%. A broad-spectrum sunscreen that protects against both ultraviolet A (UVA) and ultraviolet B (UVB) sun rays was used by only 28%. The majority (67%) did not know whether they were using broad-spectrum sunscreen or not. A water-resistant sunscreen was used by 28%. Different sunscreen types were used with cream being the most common (65%). Other sunscreen formulations included lotion (25%), spray (9%), and stick (1%). Most students (87%) consume only one bottle per month, and almost half (45%) spend 100–300 Saudi Riyals on sunscreens per month. Only 33% of students thought that commercially available sunscreens are affordable.

Regarding sun exposure duration, most of our study population (51%) are exposed to the sun 1–3 hours daily (51%), while 37% and 12% are exposed to the sun for less than 1 hour and more than 3 hours, respectively. Most (46%) get sun exposure from 10 am to 3 pm, 32% before 10 am and after 3 pm, and 22% get sun exposure all day. Reasons for sun exposure included transportation to university (81%), outdoor work or activity (45%), vitamin D (18%), leisure (15%), and sunbathing (5%). The majority of students (68%) never did sunbathing, while 30% rarely or sometimes did, and 2% often or always went sunbathing. Sunburn frequency among our population was as follows: never in 64%, one in 18%, two in 9%, three in 4%, and more than three in 5%. Family history of skin cancer was positive in approximately 1.4% of the population.

## 4. Discussion

Sunscreen application is an important method of sun protection. We have assessed sunscreen use among a large population of Saudi university students. Almost half of the students (51%) use sunscreen. In a cross-sectional study of the general population in Qassim, only 8.3% were found to use sunscreen [6]. Another cross-sectional study done in different regions of Saudi Arabia showed that approximately 24% of the general population use sunscreen [7]. Use of sunscreen among students in a Saudi university was approximately 35% [8]. The prevalence of sunscreen use in our study is much higher than previous studies conducted in Saudi Arabia. Students use sunscreen more possibly because they are younger and more educated. The higher prevalence of sunscreen use among our university students can be explained by the fact that our university teaches only health sciences. Sunscreen use prevalence in our study is similar to global rates. A study done in Brazil showed that 63% of the population use sunscreen [9]. In a study among primary and secondary school children in Switzerland, 69% of students used sunscreen [10].

Factors associated with sunscreen use in our study were female gender, high family income, sunburn history, tanning bed use, and use of other sun protection methods. Sunscreen was used more in females and people of higher social class in previous local studies [6–8]. The finding of high family income, being an independent factor for sunscreen use, is in agreement with our observation that a majority of students did not find sunscreen affordable. Top reasons for using sunscreen in the present study included prevention of sunburns and skin cancer, maintaining a light skin color, and prevention of dark spots. This was similar to the findings of Al Robaee who found that prevention of skin cancer and skin darkening to be the most common causes for using sunscreen [6]. Having a lighter skin complexion seems to be important in our community which explains the common use of sunscreen for that purpose. Inconvenience and lack of efficacy were the most common reasons for not using sunscreen in our study. Similarly, inconvenience and not believing in its importance were the main reasons for not using sunscreen in Al Robaee's study.

A majority of students use sunscreen both while in Saudi Arabia and abroad which is a good sun protection behavior that is not related to geographic areas. However, most apply sunscreen in sunny days only and mainly in summer. Studies have shown that sunscreen should also be applied in cloudy days since UV radiation is still high even in the presence of clouds and in cold weather [11, 12].

UV rays in the sun that reach the earth are divided into UVA and UVB. Both of them can cause harmful skin effects with long-term exposure [13]. Good sunscreen should have broad-spectrum protection against both UVA and UVB [14]. The majority of students in our study did not know if the sunscreen they use has broad-spectrum coverage or not. Only 28% actually used a broad-spectrum sunscreen. An SPF of >30 was used by most students (59%) in our study. This is in agreement with the current recommendations of using sunscreen with an SPF of at least 30 [14]. Standard testing of SPF in sunscreens is done using a thickness of 2 mg/cm^2^. Several studies have shown that people apply insufficient amounts of sunscreen in real life as compared to the amount recommended for SPF testing [15]. Therefore, higher SPF would compensate for this suboptimal application. In fact, several recent studies demonstrated that higher SPF sunscreens provided more protection [16–18]. Water-resistant sunscreen with a water-in-oil formulation (such as thick creams) provides better protection [14]. Although only some students used water-resistant sunscreen, a cream formulation was the most common in our study.

More than half of sunscreen users applied it less than 10 minutes before going out. The current recommendation is to apply it 15–30 minutes before sun exposure [14, 19]. Sunscreens were applied mainly to the face and hands in our study population. Approximately 30 mL of sunscreen (2-3 tablespoons) is required to effectively cover the whole body [13]. The amount of sunscreen that is considered sufficient to cover the face and neck is approximately 1-2 teaspoons [13, 14]. More than 90% of our study population applied ≤ 0.5 teaspoon. Reapplication of sunscreen is necessary every two hours or after swimming or excessive sweating [13, 19]. Most students in our study do not routinely reapply sunscreen.

The use of other sun protection methods is important. This has been shown to significantly lower the risk of sunburns [20]. Fortunately, most of our study participants used some alternative method of sun protection such as staying in shade, wearing sunglasses, long sleeves, shemagh in males, and niqab in females.

The current study has some limitations. One limitation is that our university is specialized in health sciences. This might have accounted for the relatively high prevalence of sunscreen use. Therefore, the findings in our study might not be generalizable to students in other universities. Another limitation is the lack of real-life assessment of sunscreen use. This could potentially lead to some recall bias among study participants. Performing only content validation and pilot testing for the questionnaire used in our study is a further limitation.

## 5. Conclusion

We found that half of the population in the study use sunscreen. Sunscreen was used most commonly to prevent sunburns, dark spots, and skin cancer and to maintain overall light skin color. Factors associated with sunscreen use included female gender, high family income, previous history of sunburn, use of tanning beds, and use of other sun protection methods. We have identified several areas of improper use of sunscreen. Increasing the awareness of effective sunscreen use in our community might be needed.

## Figures and Tables

**Table 1:** Subject characteristics (*n* = 1,011)^*∗*^.

Characteristic		*n*	%
Age (years), mean ± SD		21 ± 2	
Gender	Male	510	50
	Female	501	50
College year	Junior (1^st^–3^rd^ year)	530	52
	Senior (4^th^–6^th^ year)	481	48
Marital status	Single	979	97
	Married	26	3
Family income^*∗∗*^	>20,000 SR	498	52
	10,001–20,000 SR	289	30
	2,000–10,000 SR	169	18
Fitzpatrick skin phototype	I	46	5
	II	101	10
	III	290	29
	IV	412	42
	V	126	13
	VI	13	1

^*∗*^Missing data for some variables. ^*∗∗*^SR, Saudi Riyals. 1 US Dollar = 3.75 Saudi Riyals.

**Table 2:** Univariate analysis and multivariate logistic regression analysis of factors associated with sunscreen use. CI, confidence interval; NA, not applicable; OR, odds ratio.

Factor		Univariate analysis			Multivariate analysis			
		Never used sunscreen *n* (%)	Use sunscreen *n* (%)	*P* value	OR	95% CI		*P* value
Participants		496 (49%)	515 (51%)	NA	NA	NA	NA	NA
Gender	Male^*∗*^	380 (74%)	130 (26%)	**<0.001**	1			
	Female	116 (23%)	385 (77%)		10.038	7.219	13.957	**<0.001**
College year	Junior^*∗*^	260 (49%)	270 (51%)	0.998	1			
	Senior	236 (49%)	245 (51%)		0.932	0.674	1.287	0.668
Family income	2,000–10,000^*∗*^	101 (60%)	68 (40%)	**<0.001**	1			
	10,001–20,000	166 (57%)	123 (43%)		1.547	0.95	2.52	0.079
	>20,000	209 (42%)	289 (58%)		2.614	1.668	4.098	**<0.001**
Number of sunburns	None^*∗*^	368 (57%)	275 (43%)	**<0.001**	1			
	One	73 (42%)	101 (58%)		1.973	1.284	3.033	**0.002**
	Two	26 (28%)	67 (72%)		4.274	2.366	7.723	**<0.001**
	Three	14 (35%)	26 (65%)		2.133	0.947	4.802	0.067
	More than three	11 (23%)	37 (77%)		5.373	2.348	12.295	**<0.001**
Use of tanning beds	No^*∗*^	482 (51%)	468 (49%)	**<0.001**	1			
	Yes	10 (22%)	36 (78%)		2.430	1.029	5.742	**0.043**
Use of other sun protection methods	No^*∗*^	129 (66%)	66 (34%)	**<0.001**	1			
	Yes	352 (45%)	424 (55%)		1.779	1.158	2.734	**0.009**

^*∗*^Reference group.

**Table 3:** Details of sunscreen use (*n* = 515).

		*n*	%
Weather condition	Sunny days only	287	65
	Both sunny and cloudy days	152	35

Season	Summer	439	99
	Spring	211	48
	Winter	169	38
	Autumn	199	45

Place of use	Only in Saudi Arabia	47	11
	Only abroad	68	15
	Saudi Arabia and abroad	326	74

Body site	Face only	152	34
	Face and hands	186	41
	All exposed skin areas	111	25

Time of application	<10 minutes before going out	233	52
	10–20 minutes before going out	174	39
	>20 minutes before going out	39	9

Sunscreen reapplication	Every 2-3 hours	66	15
	After excessive sweating	37	8
	After swimming	59	13
	After taking a shower	91	20
	Do not reapply	278	62

Sun protection factor (SPF)	<10	6	1
	10–30	51	12
	31–50	140	32
	More than 50	119	27
	Do not know	123	28

Broad-spectrum sunscreen	Yes	123	28
	No	22	5
	Do not know	294	67

Number of bottles/tubes per month	One	373	87
	Two	38	9
	Three	14	3
	Four	3	1
	Five	1	0
	More than five	0	0

Money spent on sunscreen per month	Less than 100 SR	211	48
	100–300 SR	199	45
	301–500 SR	25	6
	501–1,000 SR	2	1
	More than 1,000 SR	0	0

## Data Availability

The data used to support the findings of this study are included within the article.

## Supplementary Materials

Supplementary Materials “Sunscreen use among a population of Saudi university students. Study questionnaire.”
