# Supplementary material for: Sunscreen Use among a Population of Saudi University Students
Source: Dermatol Res Pract. 2020 Mar 16;2020:4732721. doi: 10.1155/2020/4732721 (PMC7102484; doi:10.1155/2020/4732721)
Supplement: Supplementary Materials — “Sunscreen use among a population of Saudi university students. Study questionnaire.” [file 4732721.f1.pdf]

Sunscreen use among Saudi university students in Riyadh, Saudi Arabia

Instructions: Please put a tick ☑ in the box next to the option of your choice or write in the space provided. Please note that in **some questions you can choose more than one option**, otherwise choose only one option.

Date: ______________ **Serial Number: ______________**

1. **General information**

1.1 Age: ________ years

1.2 Gender: □ Male □ Female

1.3 College: □ COSHP □ COM □ COD □ COP □ CPHHI □ CAMS □ CON

1.4 College year:
□ First □ Second □ Third □ Fourth □ Fifth □ Sixth

1.5 Marital status: □ Single □ Married

1.6 Family monthly income in Saudi Riyals:
□ <2000 □ 2000 – 5000 □ 5001 - 10,000 □ 10,001 - 20,000 □ >20,000

2. **General questions about sunscreen use**

2.1 How often do you use sunscreen?
□ Never □ Rarely □ Sometimes □ Often □ Always

2.2 If you do not use sunscreen, what is the reason(s) for not using it?
(You can choose more than one option)
□ It is not effective
□ It is expensive
□ It is time consuming
□ It is inconvenient
□ I have sunscreen allergy
□ I have not heard about it
□ Other: ____

<u>If you do NOT use sunscreen, please skip to section 5 on page 5</u>

2.3 What is your reason(s) for using sunscreen?
(You can choose more than one option)
□ To prevent skin cancer
□ To prevent sunburns
□ To keep my skin color light
□ To prevent development of dark skin spots
□ To prevent skin wrinkling
□ Doctor recommendation
□ Family or friend recommendation
□ Social media influence
□ Read about it in the internet
□ Other: ____

2.4 When do you apply sunscreen during the day?
□ Only with outdoor activity
□ Only around noon time
□ With outdoor activity and around noon time
□ Whenever I am exposed to the sun

2.5 In which weather conditions do you use sunscreen?
□ Sunny days only □ Both sunny and cloudy days

2.6 In which season(s) do you use sunscreen?
(You can choose more than one option)
□ Summer □ Autumn □ Spring □ Winter

2.7 Where do you use sunscreen?
□ Only in Saudi Arabia □ Only abroad □ In Saudi Arabia and abroad

2.8 When you apply sunscreen, do you tend to intentionally get exposed to the sun for a longer time than when you do not apply it?
□ Yes □ No

3. Questions about sunscreen application

3.1 Which parts of your body do you usually cover by sunscreen?

- □ Face only
- □ Face and hands
- □ All exposed skin areas

3.2 How much sunscreen do you approximately apply on your face?

- □ Less than quarter teaspoon 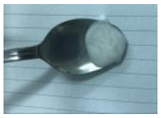
- □ Quarter to half teaspoon 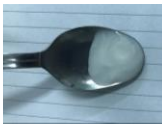
- □ More than half teaspoon 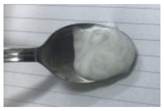

3.3 When do you usually apply sunscreen before going out?

- □ Less than 10 minutes before going out
- □ 10-20 minutes before going out
- □ More than 20 minutes before going out

3.4 When do you reapply sunscreen?
(You can choose more than one option)

- □ Every 2-3 hours
- □ After I sweat excessively
- □ After I swim
- □ After I take a shower
- □ I do not reapply it

3.5 If you want to wear makeup, when do you apply sunscreen?

- □ I do not wear makeup
- □ I do not apply sunscreen when I wear makeup
- □ Before wearing makeup
- □ After wearing makeup

4. Questions related to the type of sunscreen used and overall consumption

4.1 What is the sun protection factor (SPF) of the sunscreen you use?

□ Less than 10 □ 10-30 □ 31 - 50 □ More than 50 □ I do not know

4.2 Do you use a sunscreen that protects against both ultraviolet A (UVA) and ultraviolet B (UVB) sun rays (broad spectrum sunscreen)?

□ Yes □ No □ I do not know

4.3 Do you use a water resistant sunscreen?

□ Yes □ No □ I do not know

4.4 What sunscreen preparation do you prefer to use?

□ Cream □ Lotion □ Spray □ Stick

4.5 How many tubes (or bottles) of sunscreen do you use per month?

□ One □ Two □ Three □ Four □ Five □ More than five

4.6 How much do you spend on sunscreens monthly in Saudi Riyals?

□ Less than 100
□ 100 – 300
□ 301 – 500
□ 501 – 1000
□ More than 1000

4.7 Do you think that the commercially available sunscreens are affordable (i.e. good price that is not expensive)?

□ Yes □ No □ I do not know

5. **Questions about sun exposure and skin cancer**

5.1 What is your skin type?

(Based on skin response after exposure to the sun for half an hour)

- □ Type 1 (Always burns, never tans, very fair skin)
- □ Type 2 (Usually burns, then tans)
- □ Type 3 (May burn, tans well)
- □ Type 4 (Rarely burns, tans well)
- □ Type 5 (Very rarely burns, tans well, dark brown skin)
- □ Type 6 (Very rarely burns, tans well, very dark brown skin)

5.2 How many hours do you approximately get exposed to the sun every day?

□ Less than 1 hour □ 1-3 hours □ More than 3 hours

5.3 What is the reason(s) for sun exposure?

(You can choose more than one option)

- □ Transportation from home to university
- □ Leisure/recreational
- □ Outdoor work or activity
- □ To get enough vitamin D
- □ Sunbathing
- □ Other: ____

5.4 In which time period do you usually get exposed to the sun?

- □ 10 am to 3 pm
- □ Before 10 am or after 3 pm
- □ All day

5.5 Do you use tanning beds?

□ Yes □ No

5.6 How often do you get suntan or sunbathe?

□ Never □ Rarely □ Sometimes □ Often □ Always

5.7 How many sunburns have you had in the past?

□ None □ One □ Two □ Three □ More than three

5.8 Have you had skin cancer in the past?

□ Yes □ No

5.9 Did any family member have skin cancer in the past?

□ Yes □ No

5.10 What sun protection method(s) do you use other than sunscreen?

(You can choose more than one option)

- □ None
- □ Protective head cover (such as hijab and shomagh)
- □ Protective face cover (such as niqab and burqa)
- □ Protective body clothing (such as long sleeves and pants)
- □ Wearing sunglasses
- □ Stay in shade
- □ Using umbrella
- □ Avoiding going out during times with high sun intensity
- □ Other: ____

5.11 How often do you use sun protection methods other than sunscreen?

□ Never □ Rarely □ Sometimes □ Often □ Always

Thank you for your cooperation
